# Atypical Presentation and Unexpected Resolution: The Role of Serial Cerebrospinal Fluid Analysis in Differentiating Bacterial and Viral Meningitis After Pituitary Surgery

**DOI:** 10.7759/cureus.99802

**Published:** 2025-12-21

**Authors:** Andrea Maryant Escobar, Xavier Delgado, Ysabel Valeria Mendoza Aguilar, Manuel Vargas, Jorge Brenis Puican

**Affiliations:** 1 Faculty of Medical Sciences, Coronel y Doctor Juan Ignacio Gutiérrez Sacasa, Managua, NIC; 2 Faculty of Medicine, Espiritu Santo University, Guayaquil, ECU; 3 Faculty of Medicine, Franz Tamayo Private University, Cochabamba, BOL; 4 Faculty of Medicine, University of Aquino Bolivia, Cochabamba, BOL; 5 Faculty of Medicine, Catholic University of Santo Toribio de Mogrovejo, Chiclayo, PER

**Keywords:** antimicrobial stewardship, cerebrospinal fluid, csf fistula, differential diagnosis, viral meningitis

## Abstract

Viral meningitis can initially mimic bacterial meningitis, particularly in patients with a history of neurosurgical interventions. We report the case of a 34-year-old woman with a growth hormone (GH)/insulin-like growth factor 1 (IGF-1)-secreting pituitary tumor and multiple prior skull-base surgeries who presented with sudden-onset frontal headache. Initial cerebrospinal fluid (CSF) analysis revealed marked neutrophilic pleocytosis and elevated protein levels, prompting the initiation of empirical broad-spectrum antibiotic therapy. Despite this inflammatory profile, all cultures remained negative. Serial CSF examinations demonstrated a progressive decline in leukocyte counts with a shift toward mononuclear predominance, findings consistent with an evolving viral meningitis. Viral PCR was not available, so diagnostic interpretation relied on the clinical course and serial CSF analysis. Given the neurosurgical context and the favorable evolution of CSF parameters, antimicrobial therapy was discontinued, and antiviral treatment was maintained. Although no pathogen was identified, continuing antiviral therapy was clinically justified based on the CSF pattern and the patient’s stable condition. She improved gradually without complications. This case highlights the importance of serial CSF analysis for clarifying meningitis etiology in high-risk postoperative patients and preventing unnecessary prolonged exposure to antibiotics.

## Introduction

Meningitis is an inflammation of the meninges that cover the brain and spinal cord, most commonly caused by bacteria or viruses. Bacterial meningitis constitutes a medical emergency due to its rapid progression and high mortality when antibiotics are not started promptly. In adults, the most common causative agents are *Streptococcus pneumoniae* and *Neisseria meningitidis*, whereas in neonates, group B *Streptococcus* and *Escherichia coli *predominate. In postneurosurgical patients, meningitis is most commonly caused by *Staphylococcus aureus*, coagulase-negative staphylococci, and Gram-negative bacilli. Viral or aseptic meningitis generally follows a more benign course and is associated with enteroviruses, herpesviruses, influenza, or arboviruses [1]. Distinguishing between these etiologies can be challenging because clinical findings and initial cerebrospinal fluid (CSF) parameters often overlap.

In postoperative patients, the diagnostic value of serial CSF analyses becomes particularly important, as these evaluations provide temporal trends that help differentiate infectious etiologies when single measurements are inconclusive. Patients with structural abnormalities of the skull base, such as those who have undergone pituitary surgery or who present with secondary endocrine dysfunction, may exhibit atypical clinical presentations, and their risk of neurological complications is considerably higher [2].

Although pituitary tumors themselves do not constitute a state of immunosuppression, several factors related to their management can increase susceptibility to central nervous system infections. Postoperative anatomical changes, CSF leaks, altered meningeal integrity, and endocrine disturbances such as adrenal insufficiency may obscure classic clinical or biochemical patterns, further complicating the diagnostic process for clinicians unfamiliar with neurosurgical conditions. Likewise, postoperative patients may present additional diagnostic challenges due to pneumocephalus, healing tissue, or inflammatory debris, which can mimic bacterial processes or create ambiguity during early evaluation.

The differential diagnosis between bacterial and viral meningitis remains crucial for guiding treatment and prognosis, yet it is often difficult due to overlapping CSF characteristics. Several studies have evaluated biomarkers such as C-reactive protein, lactate, adenosine deaminase, and viral metabolites; however, none have demonstrated sufficient sensitivity or specificity for a definitive diagnosis [3].

We present the atypical clinical case of a postoperative patient whose initial CSF profile suggested a bacterial process but subsequently evolved toward findings characteristic of viral meningitis. This diagnostic transition underscores the value of serial CSF analysis in complex postoperative scenarios, where relying solely on initial results may lead to misclassification and unnecessary prolonged antibiotic therapy.

## Case presentation

A 34-year-old mestiza woman with a medical history of a pituitary neuroendocrine tumor producing growth hormone and IGF-1, recurrent tumor with an inactive CSF fistula, and central hypothyroidism presented to the emergency department six months after her most recent pituitary surgery with a one-day history of sudden-onset frontal headache. The headache was described as pulsatile, rated 8/10 in intensity, and predominantly bifrontal in location. It was associated with a single episode of vomiting that morning. The patient denied fever, neck stiffness, blurry vision, altered level of consciousness, weakness, or paresthesia. On physical examination, she was afebrile, with no focal neurological deficits and no meningeal signs; the remainder of the examination was unremarkable. Her current medications, taken due to her underlying conditions, included levothyroxine (100 mcg Monday-Thursday, 25 mcg Friday-Sunday), cabergoline (0.5 mg Monday-Thursday), levetiracetam (500 mg every 12 hours), and magnesium citrate (400 mg daily).

On hospital day 1 (day 1 of symptoms), initial laboratory tests showed leukocytosis with neutrophil predominance on complete blood count, without additional serum biochemical abnormalities (Table 1). Given her history of CSF fistula and prior skull-base surgeries, a bacterial infection was considered the leading diagnostic possibility. Empirical antibiotic therapy with cefepime (1 g IV every eight hours) and vancomycin (1 g IV every 12 hours) was initiated while a lumbar puncture was performed for CSF analysis.

**Table 1:** Laboratory results at admission and evolution Not done (value not obtained during this time point)

Parameter	At admission	Two weeks later	At discharge	Normal reference range
Hemoglobin (g/dL)	11.4 g/dL	11.4 g/dL	11.5 g/dL	12-16 g/dL
Hematocrit (%)	33%	29.8%	30.3%	37-43%
White blood cell count x10^3^/uL	19.38 x 10^3^/uL	7.2310 x 10^3^/uL	5.92 x 10^3^/uL	5-10 x 10^3^/uL
Red blood cell count x10^3^/µL	3.83 x 10^3^/µL	3.50 x 10^3^/ µL	3.59 x 10^3^/uL	4-6.3 x 10^3^/uL
Platelets	281 x 10^3^/uL	213.0 x 10^3^/uL	309.0 x 10^3^/uL	150-500 x 10^3^/uL
Neutrophils (%)	93.4%	70.0%	55.3%	55-65%
Lymphocytes (%)	3.3%	16.6%	29.2%	25-35%
Creatinine (mg/dL)	0.68 mg/dL	0.56 mg/dL	0.51 mg/dL	0.5-0.9 mg/dL
Procalcitonin (ng/mL)	0.0642 ng/mL	0.49 ng/mL	Not done	0.05-0.1 ng/mL
C-reactive protein (mg/dL)	0.73 mg/dL	6.63 mg/dL	Not done	0.5-1 mg/dL

**Figure 1:**
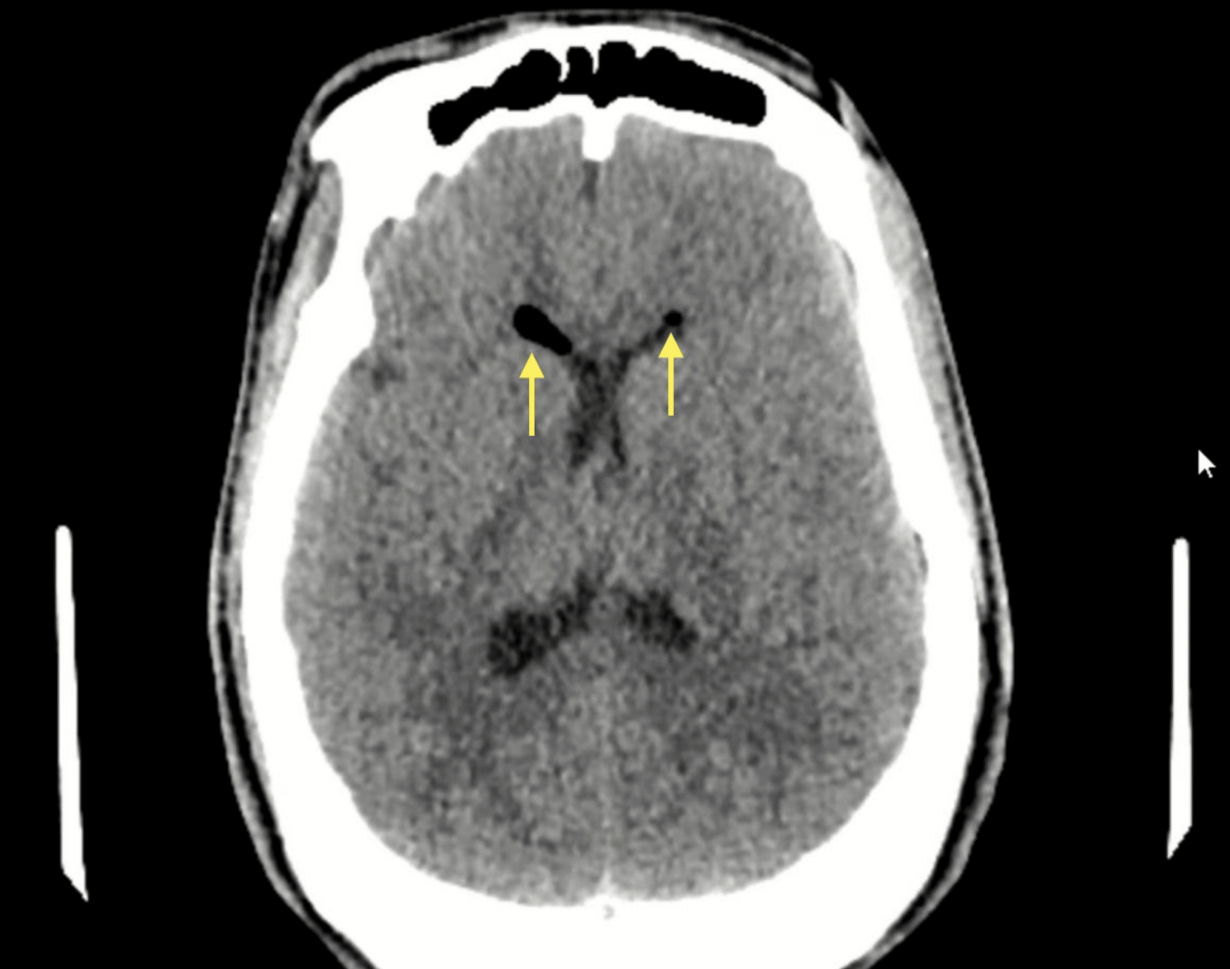
Non-contrast head CT scan Axial CT image showing minimal intraventricular pneumocephalus (yellow arrow) without evidence of hemorrhage or acute parenchymal injury.

**Figure 2:**
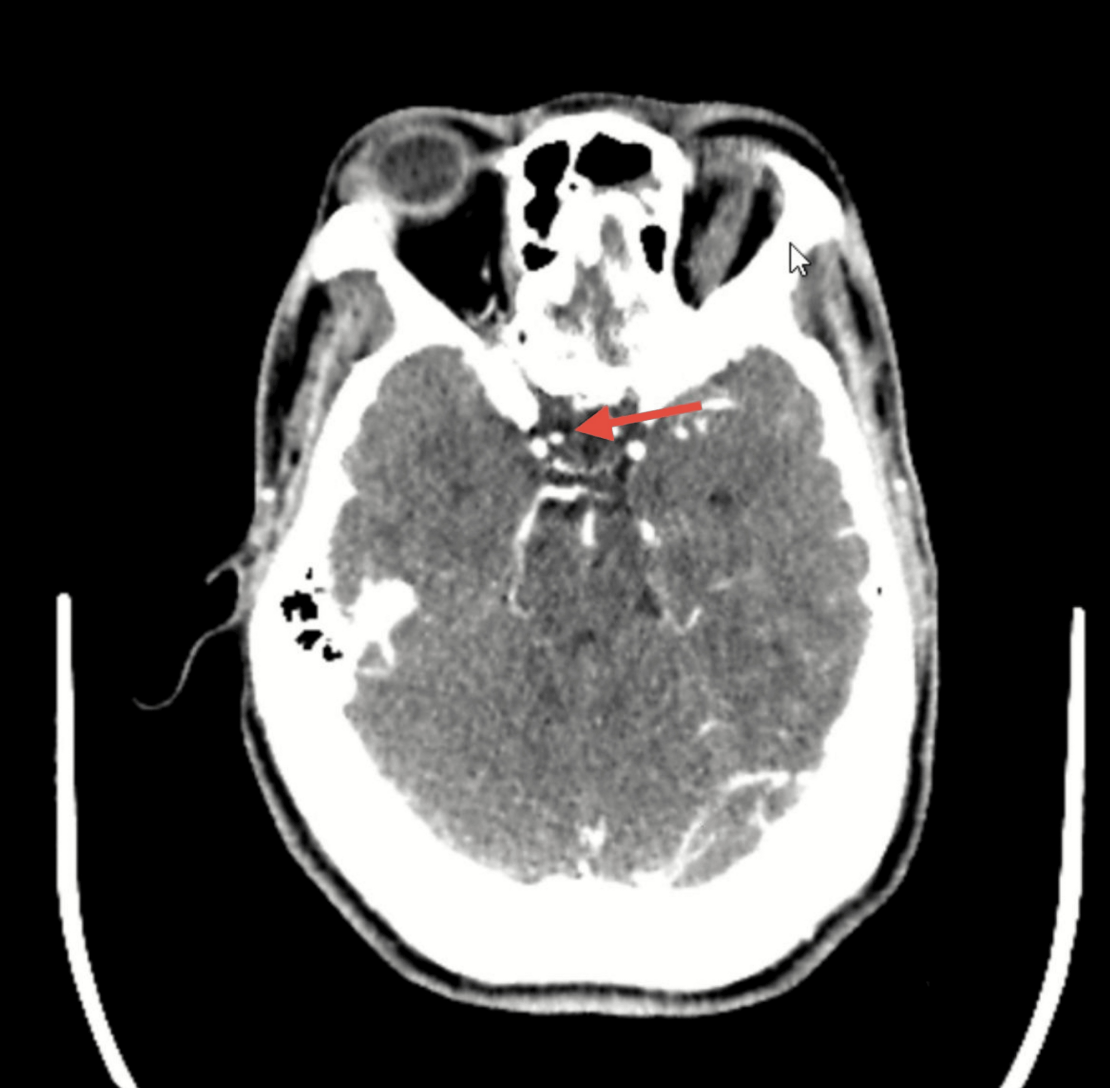
Contrast-enhanced CT angiography Axial angiographic image showing a saccular aneurysm in the clinoid segment of the right internal carotid artery (red arrow), without signs of rupture.

Computed tomography (CT) and cerebral angiography were performed on hospital day 1 due to the patient’s neurosurgical history and acute presentation. The CT scan revealed a residual postsurgical lesion in the body of the sphenoid bone, along with minimal pneumocephalus, without evidence of intracranial hemorrhage or acute parenchymal injury (Figure 1). Cerebral angiography demonstrated a saccular aneurysm in the clinoid segment of the right internal carotid artery (Figure 2). This aneurysm was deemed incidental, as the patient exhibited no symptoms or radiologic signs of aneurysmal rupture or mass effect. Therefore, it did not influence diagnostic reasoning or clinical decisions.

Subsequently, after reviewing the CSF results (Table 2) and the evaluation by the infectious disease team, antibiotic therapy was adjusted to ceftriaxone 1 g IV every 12 hours, with close clinical monitoring.

**Table 2:** Serial cerebrospinal fluid (CSF) analysis Note: "Normal CSF color" is colorless. "Clear" indicates normal transparent CSF appearence.

Parameter	At admission	Two weeks later	One month later	At discharge	Normal reference range
Color	Colorless	Colorless	Colorless	Colorless	Colorless
Appearance	Slightly turbid	Clear	Clear	Clear	Clear
Volume	4 ml	4ml	2ml	2 ml	1-5 ml
pH	8	8	8	7	7.28-7.32
Glucose (CSF)	65.3 mg/dL	65.8 mg/dL	43.7 mg/dL	47.9 mg/dL	40-70 mg/dL
Total protein	135.67 mg/dL	40.4 mg/dL	97.0 mg/dL	66.2 mg/dL	15-45 mg/dL
Leukocytes (cells/mm^3^)	2458 cells/mm^3^	13 cells/mm^3^	58 cells/mm^3^	13 cells/mm^3^	0-5 cells/mm^3^
CSF albumin	60.08 mg/dL	19.6 mg/dL	46.6 mg/dL	30.8 mg/dL	15-45 mg/dL
Polymorphonuclear (%)	92.4%	7%	1%	2%	0-6%
Mononuclear cells (%)	7.6%	92%	98%	100%	0-6%
Gram stain	Negative	Negative	Negative	Negative	Negative
Direct Koh (fungal)	Negative	Negative	Negative	Negative	Negative
CSF culture	No growth	No growth	No growth	No growth	No growth

A follow-up CSF study was scheduled two weeks later. At that time, an increase in serum C-reactive protein was observed. The repeat lumbar puncture demonstrated a significant decrease in leukocytes; however, mononuclear cells remained elevated, consistent with a viral infection pattern. For this reason, antiviral therapy was added despite the absence of associated symptoms. A macrolide was also included to provide coverage for atypical pathogens, and oral acyclovir was initiated at 800 mg every eight hours.

Because viral PCR testing was not available and the patient remained clinically stable without features suggestive of HSV or VZV infection, such as mucocutaneous lesions, altered mental status, or focal neurological deficits, oral acyclovir was continued rather than escalating to intravenous therapy. This decision was guided by the serial CSF evolution, which showed a clear transition toward a mononuclear viral pattern with persistently negative cultures.

One week after this, another lumbar puncture revealed resolution of the bacterial process, with mild pleocytosis composed predominantly of mononuclear cells and slight hyperproteinorrachia, all trending downward compared to the previous CSF analysis. With no abnormalities found in special CSF stains, antibiotic therapy was discontinued. In the final lumbar puncture, findings were consistent with viral meningitis in the resolution phase.

During hospitalization, the patient remained afebrile, without new neurological signs, with preserved renal function, and without criteria for systemic inflammatory response syndrome. She was discharged with a diagnosis of resolving viral meningitis, continuing oral acyclovir (400 mg every eight hours for 15 days), supportive analgesia, and follow-up with Internal Medicine and Neurosurgery.

## Discussion

The present case describes a 34-year-old woman with a complex medical history, including a pituitary neuroendocrine tumor producing growth hormone and insulin-like growth factor 1, multiple skull-base surgeries, and an inactive CSF fistula. The patient presented with a sudden-onset frontal headache without fever or meningeal signs, but with leukocytosis and an inflammatory initial CSF profile, which initially raised suspicion for bacterial meningitis. This case is clinically relevant because it exemplifies the diagnostic and therapeutic difficulty in distinguishing between bacterial and viral meningitis in a postoperative neurosurgical context, where the risk of central nervous system infections is elevated, and the margin for therapeutic error is narrow.

Likewise, in patients with prior skull-base surgery and meningeal anatomical alterations, alternative diagnoses that may mimic true meningeal infection must be considered. These include chemical meningitis, which may result from meningeal irritation secondary to blood products, surgical materials, or substances introduced during procedures; sterile postoperative inflammation, which can produce pleocytosis and elevated protein levels in the absence of microorganisms; and drug-induced meningitis, which has been described with agents such as nonsteroidal anti-inflammatory drugs, immunoglobulins, trimethoprim-sulfamethoxazole, and certain antibiotics. These processes may present with clinical and CSF findings similar to bacterial meningitis, making interpretation of a single CSF analysis insufficient. In this context, serial CSF evaluation, correlation with prior interventions, and systematic exclusion of pathogens through cultures and molecular testing are essential to guide diagnosis and establish appropriate management.

The main clinical challenge was determining the etiology of the meningeal process. In practice, distinguishing between bacterial and viral meningitis can be complex, even when laboratory tests and CSF studies are available. According to the literature, bacterial meningitis typically presents with polymorphonuclear-predominant pleocytosis, hypoglycorrhachia, and markedly elevated protein levels; however, up to 10-20% of viral meningitis cases may initially show a bacterial-like profile, with neutrophil predominance during the first 24-48 hours of clinical presentation [4,5]. In this case, the initial CSF analysis showed marked leukocytosis (2458/mm³, 92% polymorphonuclear cells) and elevated protein levels, findings suggestive of bacterial infection. Nevertheless, cultures were negative, and subsequent evolution demonstrated a shift toward a mononuclear pattern with progressive decline in leukocytes and protein levels, consistent with a viral etiology.

Interpretation of serial CSF parameters requires caution in postoperative patients, in whom inflammatory markers and protein concentrations may fluctuate due to blood-CSF barrier permeability changes and tissue healing. The increase in CSF protein observed at the one-month follow-up (from 40.4 to 97.0 mg/dL) warrants careful interpretation. In postoperative patients, protein levels may rise transiently due to ongoing meningeal healing, residual inflammatory remodeling, or temporary alterations in barrier permeability, even when the infectious process is resolving. Importantly, this isolated protein elevation occurred in the context of continued leukocyte decline, mononuclear predominance, persistently negative cultures, and stable clinical status, findings that do not support recurrent or persistent infection. Accordingly, this fluctuation was interpreted as a postoperative or inflammatory variation rather than evidence of disease progression.

The CSF pH value of 8.0 reported in this case is unusually high and was interpreted as likely representing a measurement artifact. CSF pH is not routinely assessed using standardized laboratory techniques, and exposure of the sample to ambient air or delays in processing may artificially elevate values. Importantly, no clinical features or accompanying CSF abnormalities supported a truly alkaline pH; therefore, this finding was not considered diagnostically meaningful.

The diagnostic difficulty was further heightened by the patient’s surgical and anatomical history, which increases the risk of bacterial infections of the central nervous system. Prior skull-base surgeries and the presence of a CSF fistula, even if inactive, increase the theoretical risk of recurrent bacterial meningitis caused by pathogens originating from the nasosinusal tract. An inactive CSF fistula is clinically defined as the absence of overt leakage-without rhinorrhea, positional dripping, or endoscopic evidence of an open communication-despite a documented history of a prior osteodural defect [6]. Although such fistulas do not demonstrate active leakage, several studies indicate that any residual structural disruption of the meningeal barrier may maintain an increased risk of infection, particularly in patients with repeated surgeries or partial skull-base reconstructions [7-9]. In this case, computed tomography imaging demonstrated postoperative changes involving the sphenoid body and minimal pneumocephalus, findings that suggest residual anatomical fragility despite the absence of evidence of an active ongoing fistula. This clinical context justified the initiation of empirical broad-spectrum antibiotic therapy, supported by international guidelines for the treatment of central nervous system infections in patients with prior neurosurgical history.

The interpretation of CSF is crucial in the diagnosis of viral meningitis, as initial findings may not reflect the true etiology. Early predominance of polymorphonuclear cells can mimic bacterial infection; therefore, diagnosis should not rely on a single lumbar puncture. Repeat lumbar puncture may be considered when diagnostic uncertainty persists, when patients worsen after 48 hours of antibiotic treatment, or when there is no clinical improvement, with the aim of ruling out reinfection and guiding antimicrobial duration [10,11]. However, no recent studies clearly define the optimal indication or interval for repeated lumbar punctures in adults.

Viral diagnosis may be supported by serological studies, including detection of specific immunoglobulin M, immunoglobulin G seroconversion, or intrathecal antibodies, as well as nucleic acid amplification techniques, which offer high sensitivity and rapid results and currently represent the preferred method for pathogen identification in CSF [12]. In this context, serial studies can support safer and more precise therapeutic management, particularly when initial findings are ambiguous.

The therapeutic approach was appropriate, as the patient presented with a sudden-onset headache, a history of an inactive CSF fistula, and leukocytosis, raising initial suspicion for bacterial infection. Empirical treatment with cefepime and vancomycin was initiated, followed by de-escalation to ceftriaxone. According to international recommendations, antimicrobial therapy and corticosteroids should be initiated immediately in suspected bacterial meningitis, even when viral etiology remains possible [13]. After bacterial infection was excluded and serial CSF studies showed a mononuclear shift, viral meningitis was suggested, antibiotics were discontinued, and treatment was continued with acyclovir alone. Although intravenous acyclovir is recommended when meningitis caused by herpes simplex virus or varicella zoster virus is clinically suspected, this patient did not exhibit features suggestive of these infections and remained clinically stable. Given the absence of viral polymerase chain reaction testing and the progressive mononuclear shift observed on serial CSF analyses, oral acyclovir was considered an appropriate therapeutic option.

The absence of an identified viral pathogen reduces diagnostic certainty and may lead to repeated procedures or prolonged therapies; however, the favorable clinical evolution, improvement in CSF parameters, and follow-up imaging supported the adequacy of the management strategy. Imaging demonstrated progressive resolution of the previously observed pneumocephalus without associated complications. The saccular aneurysm in the cavernous segment of the right internal carotid artery remained stable, without evidence of rupture. This case highlights the importance of early empirical therapy combined with ongoing reassessment based on clinical evolution, laboratory findings, and imaging, allowing rational antimicrobial use once a viral etiology is supported.

## Conclusions

Patients with a history of pituitary surgery have an increased risk of meningeal infections and require careful diagnostic evaluation when presenting with compatible symptoms. In this case, serial lumbar punctures were essential for monitoring the dynamic evolution of the CSF, allowing timely differentiation of a resolving viral process despite initially ambiguous findings. This strategy prevented unnecessary prolongation of antibiotic therapy and enabled treatment to be adjusted according to the actual CSF trends, supporting responsible antimicrobial stewardship in the postoperative setting. Serial CSF assessments proved critical for guiding clinical decisions in scenarios where early laboratory results may be misleading, highlighting their value in complex postoperative patients.
